# Vertebral Bone Quality (VBQ) as a Novel MRI-Based Assessment Tool: Is It an Alternative to the Time-Tested Dual-Energy X-ray Absorptiometry (DEXA) Scan?

**DOI:** 10.7759/cureus.97983

**Published:** 2025-11-27

**Authors:** Sameer J Ahamed, Mahesh Shekoba, Ankith N V, Sabah Sulaiman, Aneesh MK, Monish Nachu, Srinivasalu Santhanagopal, Mallikarjuna B Swamy

**Affiliations:** 1 Department of Spine Surgery, St. John’s Medical College Hospital, Bangalore, IND

**Keywords:** bone mineral density, ct, dexa, hounsfield units, mri, osteoporosis, vertebral bone quality

## Abstract

Purpose

To compare CT-derived Hounsfield units (HU) and MRI-based vertebral bone quality (VBQ) scores with dual-energy X-ray absorptiometry (DEXA), and to evaluate their diagnostic performance for identifying osteoporosis.

Overview of the literature

DEXA is the gold standard for assessing bone mineral density (BMD), but it has limitations, including radiation exposure and interference from degenerative changes. HU and VBQ are promising, radiation-free imaging techniques assessing different aspects of bone health: HU reflects trabecular bone density, while VBQ quantifies marrow fat content, inversely related to bone strength.

Methods

This study is a retrospective analysis of 112 adults who underwent lumbar spine DEXA, CT, and MRI within 30 days. HU was measured at the L3 vertebral body on CT, and VBQ scores were derived from MRI by comparing vertebral signal intensities to cerebrospinal fluid. Correlations with DEXA T-scores and diagnostic performance metrics, including receiver operating characteristic (ROC) analysis, were assessed.

Results

HU values demonstrated a positive correlation with DEXA T-scores (r = 0.549, p < 0.001), with decreasing HU values as BMD worsened. VBQ scores showed a weaker, non-significant correlation (r = -0.175, p = 0.064). HU exhibited higher diagnostic accuracy (area under the curve (AUC) = 0.90) than VBQ (AUC = 0.75). Combination testing improved diagnostic performance: series testing (both HU ≤ 110 and VBQ ≥ 3.9) showed 50% sensitivity and 83% specificity, while parallel testing (either HU ≤ 110 or VBQ ≥ 3.9) demonstrated 86% sensitivity and 51% specificity.

Conclusions

CT-derived HU and MRI-based VBQ scores are complementary biomarkers for osteoporosis assessment. HU offers strong diagnostic accuracy, while VBQ provides a radiation-free alternative. The combination of both methods enhances diagnostic performance, making them valuable for osteoporosis screening, especially when DEXA is unavailable.

## Introduction

Osteoporosis is a skeletal disorder marked by low bone mass and microarchitectural deterioration, increasing fracture risk [1,2]. Early diagnosis is vital to prevent fragility fractures, especially in the elderly. Dual-energy X-ray absorptiometry (DEXA) remains the gold standard for assessing bone mineral density (BMD) and guiding management with the WHO-based T-scores categorizing patients as normal, osteopenic, or osteoporotic [3].

Despite its widespread use, DEXA has several important limitations. First, it provides only a two-dimensional areal BMD measurement that lacks information about bone microarchitecture and quality [3,4]. Second, DEXA results may be skewed by degenerative changes, aortic calcifications, and vertebral fractures, particularly in the elderly [5]. Additionally, accessibility and patient compliance with DEXA screening remain suboptimal in certain clinical settings with added risk of radiation exposure [6].

Given these limitations, newer imaging modalities are being studied as alternatives for bone health assessment. Vertebral bone quality (VBQ) is a magnetic resonance imaging (MRI)-based technique that quantifies marrow fat by comparing vertebral signal intensity to cerebrospinal fluid on T1-weighted images [7]. Higher VBQ scores, indicating increased marrow fat, are inversely associated with bone strength [8].

Ehresman et al. demonstrated a strong correlation between VBQ scores and DEXA T-scores, validating VBQ as a surrogate marker for osteoporosis [7]. The key advantage of VBQ is that it is radiation-free, opportunistically applicable on routine spinal MRIs, and especially useful in patients undergoing MRI for back pain, neurological symptoms, or surgical planning [7,8]. Moreover, it allows segmental analysis of bone quality, which is particularly relevant in spine surgery planning.

Hounsfield units (HU) from computed tomography (CT) scans correlate with trabecular bone density and are increasingly used for opportunistic screening in trauma, oncology, and spine surgery patients [9]. Schreiber et al. defined HU thresholds for osteoporosis detection with good sensitivity and specificity, showing strong agreement with DEXA [10]. Like VBQ, HU can be extracted from routine scans, improving cost-effectiveness and accessibility [11]. Although CT involves radiation, it is often obtained preoperatively, making HU a practical opportunistic tool. HU reflects bone density but not marrow composition, whereas VBQ provides a more comprehensive, radiation-free assessment, suitable for repeated use and in younger or radiation-sensitive patients [8,12,13].

This study investigates whether VBQ and HU can complement or replace DEXA, particularly when DEXA is unavailable or unreliable, by evaluating their diagnostic value as practical, non-invasive tools for vertebral bone health.

## Materials and methods

Study design and setting

This retrospective study was approved by the Institutional Ethics Committee (Approval No.: IEC/1/1313/2025). As it was retrospective, informed consent was waived in accordance with institutional guidelines. This study involved human patient data and was conducted in accordance with institutional ethical standards.

Study objective

This study aimed to compare CT-derived HU and MRI-based VBQ scores with DEXA, and to determine their diagnostic accuracy in osteoporosis detection. We hypothesized that HU would show a stronger correlation with DEXA T-scores than VBQ, and that combining HU and VBQ thresholds would improve diagnostic accuracy compared with either parameter alone.

Patient selection

The study included 112 adults who underwent lumbar spine MRI, CT, and DEXA within 30 days to minimize BMD variation. Exclusion criteria were prior spinal surgery, spinal infection, tumors, other metabolic bone diseases, or severe lumbar degeneration (e.g., extensive osteophytes in ≥3 vertebrae, terminal disc degeneration, or ≥3 adjacent facet joint narrowing with large osteophytes), as these could distort imaging assessments [14].

Bone mineral density assessment via DEXA

BMD was measured using the GE Lunar Prodigy Advance (GE Healthcare, Chicago, IL). The lowest T-score from the lumbar spine, femoral neck, or total hip was classified per the WHO criteria: osteoporosis (≤−2.5), osteopenia (−2.5 to −1), and normal (≥−1) [15]. Lumbar spine DEXA values from eligible patients were used as the reference standard [16-18].

HU value measurement from CT

**Figure 1:**
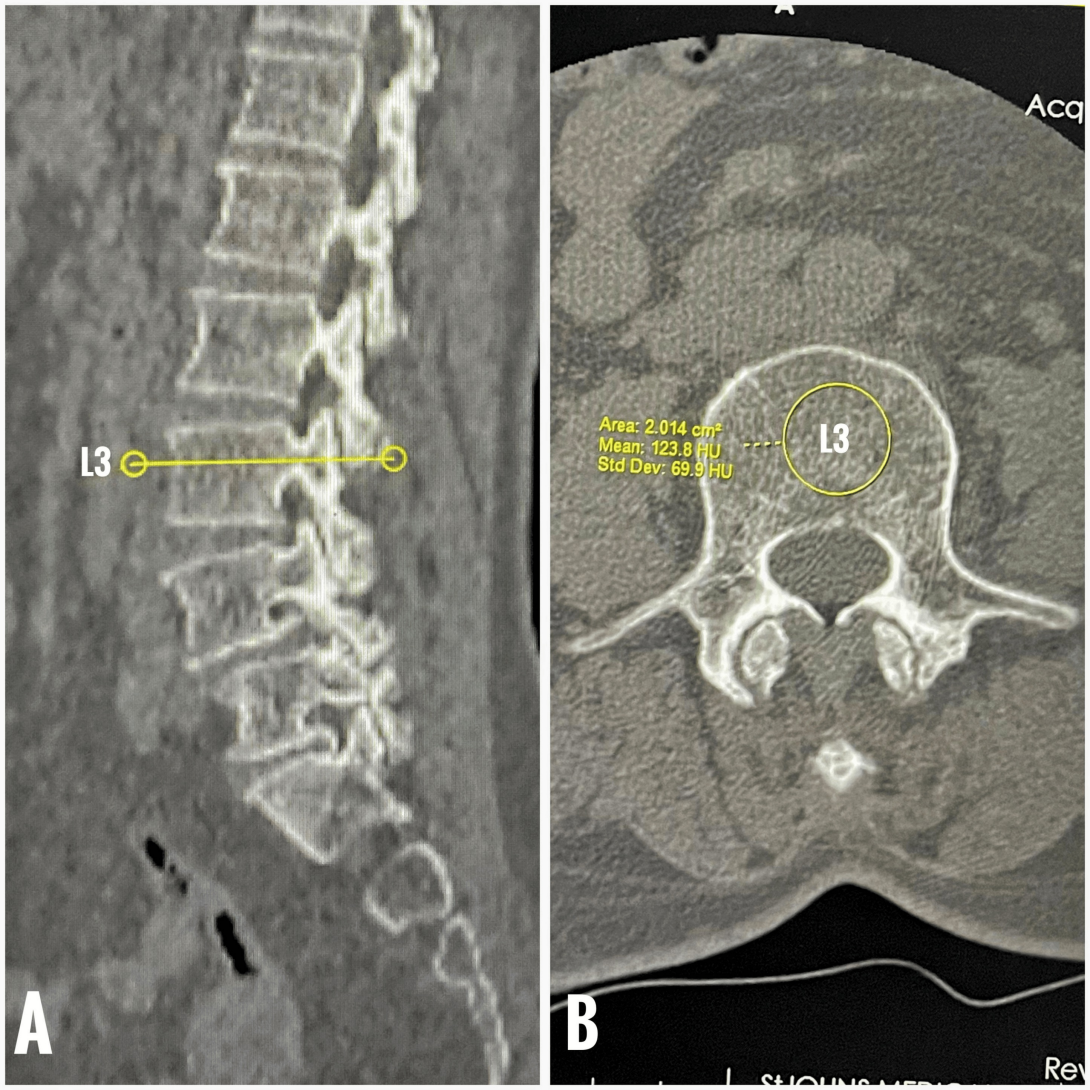
Opportunistic quantification of vertebral trabecular density using CT-derived Hounsfield unit (HU) analysis at mid-vertebral level L3.

HU values were measured on axial lumbar CT images using PACS (Picture Archiving and Communication System). CT scans were obtained using a Siemens (Erlangen, Germany) model with standard lumbar parameters (120 kVp, 3-5 mm slice thickness, non-contrast protocol). These acquisition settings are included to enhance reproducibility. Elliptical regions of interest (ROIs) were placed at the L3 mid-vertebral body, targeting cancellous bone while avoiding cortical margins, sclerosis, fractures, or focal lesions (Figure 1). This approach, validated in prior studies, shows good correlation with DEXA T-scores and vertebral compressive strength [4,9,10,19-25].

VBQ score measurement from MRI

**Figure 2:**
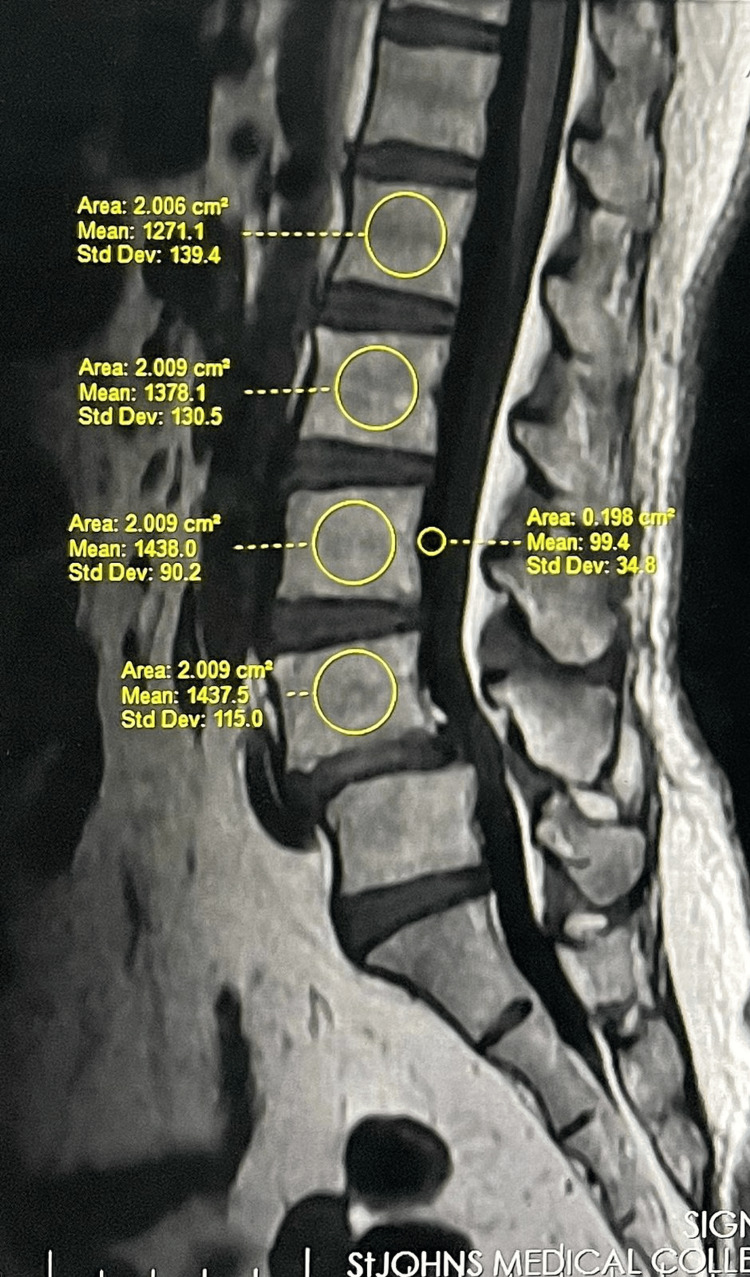
MRI-based vertebral bone quality (VBQ) score derivation from routine T1-weighted sagittal non-contrast sequences.

VBQ scores were derived from sagittal T1-weighted lumbar MRI. MRI was performed on a 3-Tesla scanner using sagittal T1-weighted spin-echo sequences with TR (repetition time) = 500-700 ms, TE (echo time) = 8-12 ms, slice thickness = 3 mm, interslice gap = 0.3 mm, field of view = 300-340 mm, and matrix = 256 × 320. ROIs were placed in vertebral bodies L1-L4 on the mid-sagittal slice; parasagittal slices were used if anomalies (e.g., hemangiomas and venous plexus) were present. Vertebrae with diffuse abnormalities or fractures were excluded. The median signal intensity (SI) of L1-L4 was divided by CSF SI at L3 (or L2 if L3 CSF was unclear) to obtain the VBQ score (Figure 2) [7,26-28]. We acknowledge that CSF signal variability may influence VBQ calculation; however, standardized ROI placement and consistent sequence parameters minimized this effect. VBQ score = Median SI (L1-L4)/SI of CSF at L3 level.

To ensure consistency, HU values and VBQ scores were independently measured by the primary investigator and a trained radiologist, showing strong agreement between observers.

Statistical analysis

Demographic variables were analyzed to avoid bias from age or gender. Homogeneity of HU and VBQ data across groups (normal, osteopenia, osteoporosis) was tested with Levene’s statistic. ANOVA was used when data were homogeneous; otherwise, the Kruskal-Wallis test was applied. Categorical variables were compared using chi-square or Fisher’s exact test. Pearson’s correlation assessed relationships between HU, VBQ, and BMD. For receiver operating characteristic (ROC) analysis, osteopenia and osteoporosis groups were combined to compare normal vs. osteoporotic, and thresholds were established based on sensitivity and specificity. Analyses were performed using SPSS v19 (IBM Corp., Armonk, NY).

## Results

The final cohort included 112 patients, comprising 44 males (39.3%) and 68 females (60.7%), with a mean age of 60.25 ± 11.98 years. Based on DEXA, 41 patients (36.6%) had normal BMD, 38 (33.9%) had osteopenia, and 33 (29.5%) had osteoporosis. Mean ages were 57.95 ± 11.88 years (normal), 60.79 ± 12.25 years (osteopenia), and 62.48 ± 11.64 years (osteoporosis). One-way ANOVA showed no significant difference in mean age across groups (p = 0.257) (Table 1).

**Table 1:** Age distribution among normal, osteopenia, and osteoporotic patients as classified by DEXA criteria. DEXA = dual-energy X-ray absorptiometry.

Group	N	Mean	Std. deviation	Minimum	Maximum	P-value
Normal	41	57.95	11.878	33	79	0.257
Osteopenia	38	60.79	12.252	21	80	
Osteoporosis	33	62.48	11.638	32	80	
Total	112	60.25	11.978	21	80	

In the normal BMD group, there were 23 females and 18 males; in osteopenia, 22 females and 16 males; and in osteoporosis, 23 females and 10 males. Chi-square analysis showed no significant association between gender and BMD group (χ² = 0.92, df = 2, p = 0.631; Cramer’s V = 0.09) (Table 2).

**Table 2:** Gender stratification across DEXA-defined BMD categories. DEXA = dual-energy X-ray absorptiometry; BMD = bone mineral density.

	Gender			
	Female	Male	Total	P-value
Normal	23	18	41	0.631
	56.10%	43.90%	100.00%	
	33.30%	41.90%	36.60%	
Osteopenia	24	14	38	
	63.20%	36.80%	100.00%	
	34.80%	32.60%	33.90%	
Osteoporosis	22	11	33	
	66.70%	33.30%	100.00%	
	31.90%	25.60%	29.50%	
Total	69	43	112	
	61.60%	38.40%	100.00%	
	100.00%	100.00%	100.00%	

Mean DEXA spine T-scores declined across BMD categories: normal = 0.229 ± 1.07, osteopenia = -1.85 ± 0.45, and osteoporosis = -3.23 ± 0.60. Interobserver reliability was excellent, with intraclass correlation coefficients (ICCs) of 0.91 for HU and 0.89 for VBQ.

**Figure 3:**
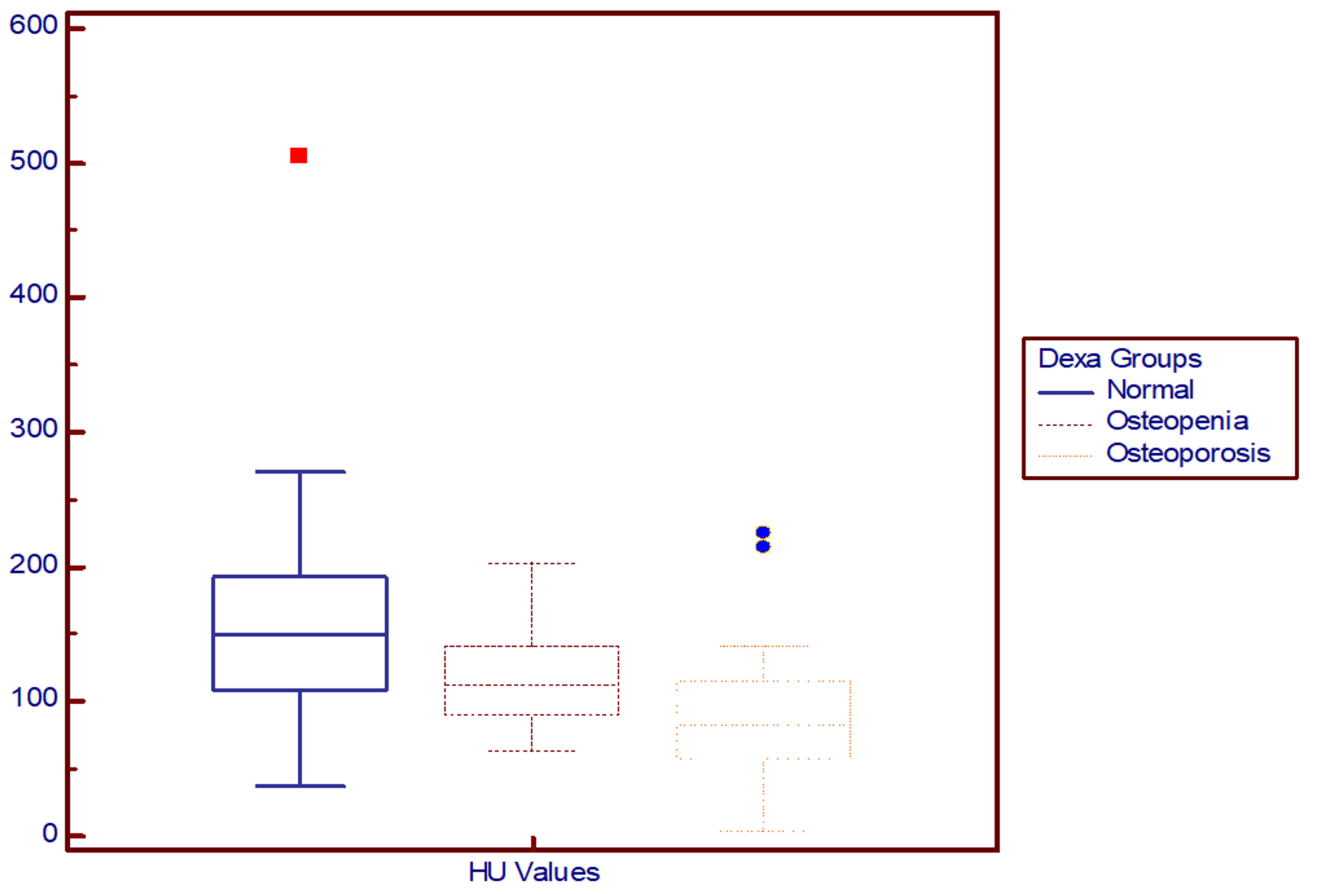
Box-and-whisker plot demonstrating the distribution of CT-derived vertebral HU values across DEXA-classified BMD categories. CT = computed tomography; HU = Hounsfield unit; DEXA = dual-energy X-ray absorptiometry; BMD = bone mineral density.

HU values declined with worsening BMD, with significant differences: 158.8 ± 77.6 (normal), 117.2 ± 40.3 (osteopenia), and 87.9 ± 49.0 (osteoporosis). This downward trend was statistically significant (p < 0.001). CT-derived HU values thus effectively distinguished between BMD categories (Figure 3).

**Figure 4:**
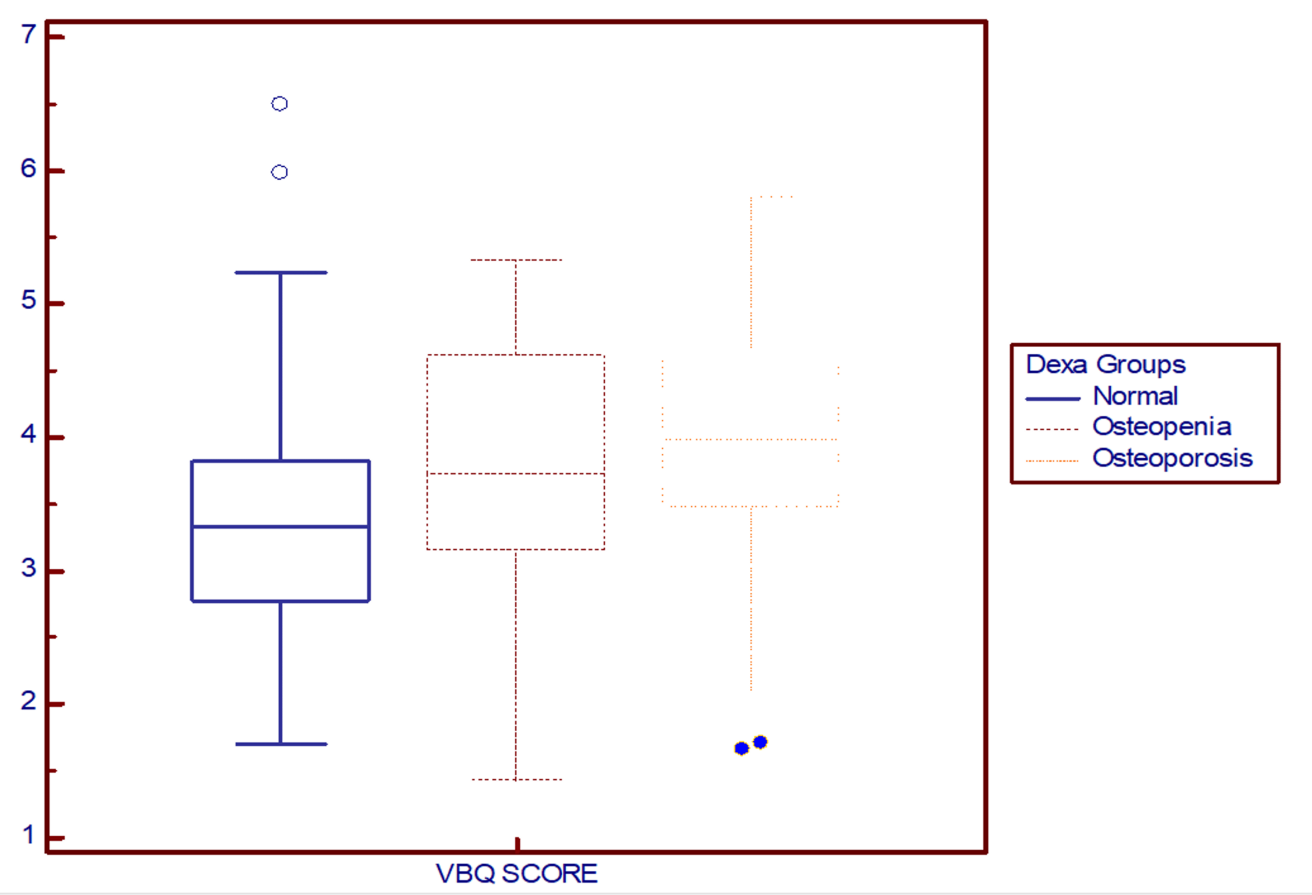
Box-and-whisker plot depicting the distribution of MRI-derived VBQ scores across BMD subgroups. MRI = magnetic resonance imaging; VBQ = vertebral bone quality; BMD = bone mineral density.

VBQ scores increased with worsening BMD, showing a significant trend (p = 0.047) (Table 3): 3.43 ± 1.01 (normal), 3.76 ± 1.00 (osteopenia), and 4.02 ± 1.07 (osteoporosis). MRI-derived VBQ thus showed stepwise differentiation across BMD categories (Figure 4).

**Table 3:** Descriptive and inferential statistics for DEXA T-scores, HU values, and VBQ scores across BMD categories. DEXA = dual-energy X-ray absorptiometry; HU = Hounsfield unit; VBQ = vertebral bone quality; BMD = bone mineral density.

Variable	Group	N	Mean	Std. deviation	Minimum	Maximum	p-value
DEXA spine	Normal	41	0.229	1.068	-1	3.6	<0.001
	Osteopenia	38	-1.847	0.4464	-2.4	-1.1	
	Osteoporosis	33	-3.233	0.604	-5.2	-2.5	
	Total	112	-1.496	1.6192	-5.2	3.6	
VBQ score	Normal	41	3.4272	1.0142	1.7	6.5	0.047
	Osteopenia	38	3.7632	1.001	1.43	5.33	
	Osteoporosis	33	4.0246	1.0716	1.67	5.81	
	Total	112	3.7172	1.0467	1.43	6.5	
HU - All	Normal	41	158.785	77.599	37.9	505.5	<0.001
	Osteopenia	38	117.224	40.3307	63.4	203	
	Osteoporosis	33	87.876	48.9614	3.2	225	
	Total	112	123.791	65.2212	3.2	505.5	

Pearson correlation showed HU had a moderate positive association with DEXA spine T-scores (r = 0.549, p < 0.001), indicating higher HU with better BMD. VBQ demonstrated a weak, non-significant negative correlation (r = -0.175, p = 0.064), suggesting limited direct association with DEXA in this cohort (Table 4).

**Table 4:** Bivariate correlation matrix between imaging parameters (HU and VBQ) and DEXA spine T-scores. DEXA = dual-energy X-ray absorptiometry; HU = Hounsfield unit; VBQ = vertebral bone quality.

		DEXA spine	HU - All	VBQ score
DEXA spine	Pearson correlation	1	0.549	-0.175
	Sig. (2-tailed)		0.000	0.065
	N	112	112	112

Both HU values and VBQ scores demonstrated potential as imaging markers for osteoporosis detection, but their diagnostic performance varied.

An HU threshold of ≤ 123 yielded 68% sensitivity, 70% specificity, and a Youden index = 0.387, indicating moderate diagnostic performance. A stricter threshold of ≤ 110 improved diagnostic ability, with sensitivity ≈ 85-90%, specificity ≈ 80-85%, and AUC = 0.748 (95% CI: 0.654-0.842). These findings are summarized in Tables 5, 6.

**Table 5:** Diagnostic threshold analysis of CT-derived Hounsfield unit (HU) values for osteoporosis detection (AUC results). AUC = area under the curve.

Parameter	AUC	Std. error	95% confidence interval
HU (Hounsfield unit)	0.748	0.048	0.654 – 0.842

**Table 6:** Diagnostic threshold analysis of CT-derived Hounsfield unit (HU) values for osteoporosis detection (sensitivity and specificity).

Threshold (HU)	Sensitivity (%)	Specificity (%)	Youden index
≤123	68	70	0.387

VBQ values increased with worsening bone status. A threshold of ≥ 3.5 achieved 72% sensitivity, 66% specificity, Youden index = 0.377, and AUC = 0.659 (95% CI: 0.553-0.764). A stricter cutoff of ≥ 3.9 yielded sensitivity ≈ 70-75% and specificity ≈ 65-70% (Tables 7, 8).

**Table 7:** Diagnostic threshold analysis of MRI-derived VBQ scores for osteoporosis detection (AUC results). MRI = magnetic resonance imaging; VBQ = vertebral bone quality; AUC = area under the curve.

Parameter	AUC	Std. error	95% confidence interval
VBQ	0.659	0.054	0.553 – 0.764

**Table 8:** Diagnostic threshold analysis of MRI-derived VBQ scores for osteoporosis detection (sensitivity and specificity). MRI = magnetic resonance imaging; VBQ = vertebral bone quality.

Threshold (VBQ)	Sensitivity (%)	Specificity (%)	Youden index
≥3.5	72	66	0.377

In series combination (HU ≤110 and VBQ ≥3.9), AUC was 0.689 (95% CI: 0.590-0.789), with 50% sensitivity, 83% specificity, and a Youden index of 0.33. In parallel (either HU ≤110 or VBQ ≥3.9), AUC was 0.686 (95% CI: 0.578-0.793), with 86% sensitivity, 51% specificity, and a Youden index of 0.37. Both were significant (p = 0.001) (Tables 9, 10), suggesting series testing better confirms osteoporosis, while parallel testing improves screening.

**Table 9:** Comparative summary of diagnostic performance metrics for HU and VBQ thresholds. HU = Hounsfield unit; VBQ = vertebral bone quality; AUC = area under the curve.

Parameter	Cut-off value	Sensitivity (%)	Specificity (%)	AUC (approx.)	p-value
VBQ	≥3.9	72.5%	67.5%	0.75	0.005
HU	≤110	87.5%	82.5%	0.90	<0.001

**Table 10:** Diagnostic performance metrics of series and parallel combinations of HU and VBQ for osteoporosis detection. HU = Hounsfield unit; VBQ = vertebral bone quality; AUC = area under the curve.

Combination Type	AUC (95% CI)	Std. Error	p-value	Sensitivity (%)	Specificity (%)	Youden Index
Series	0.689 (0.590–0.789)	0.051	0.001	50	83	0.33
Parallel	0.686 (0.578–0.793)	0.055	0.001	86	51	0.37

**Figure 5:**
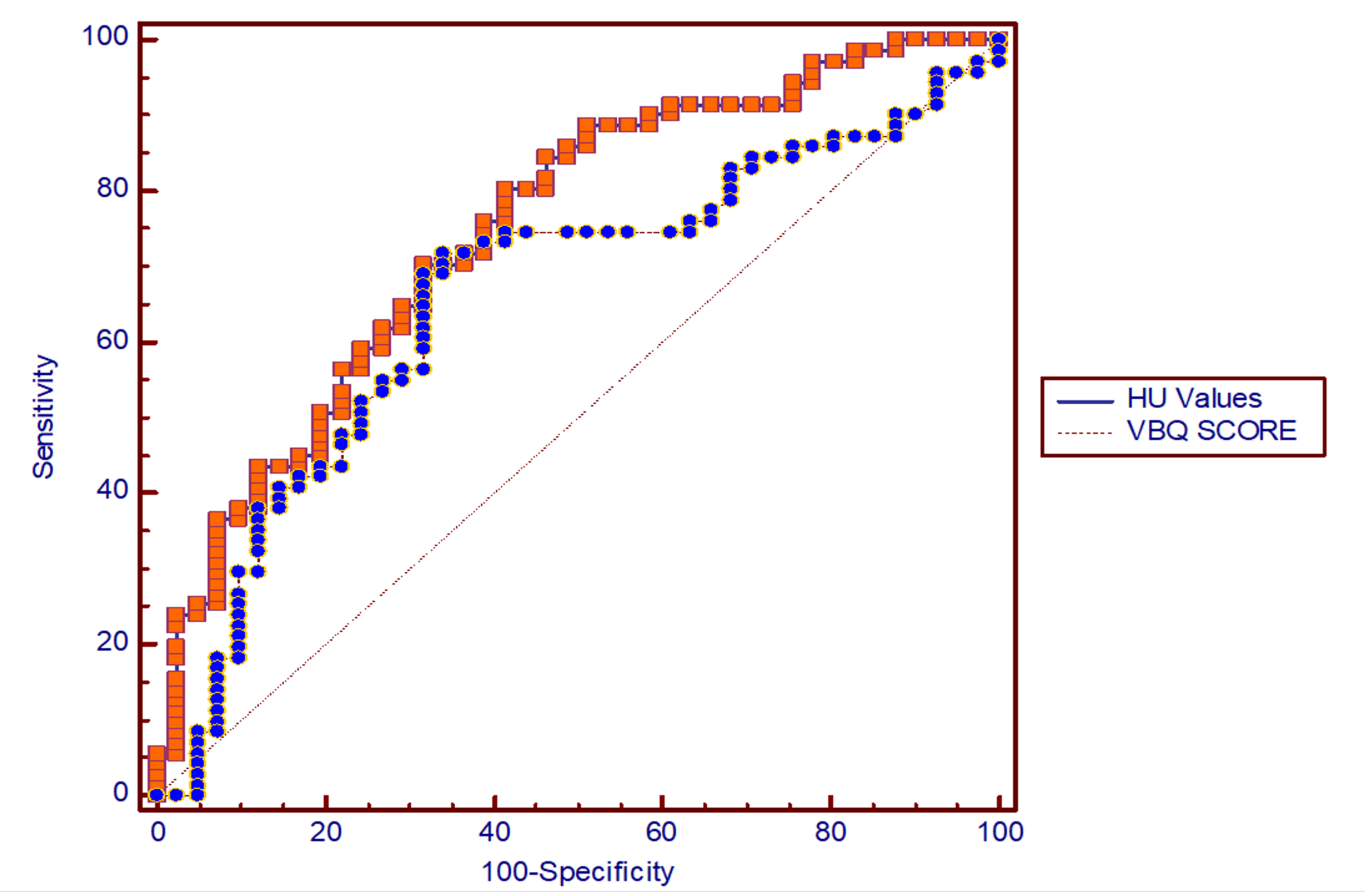
Receiver operating characteristic (ROC) curve (threshold-performance diagnostic modeling)-based comparative diagnostic modeling of CT-derived HU values and MRI-derived VBQ scores in osteoporosis detection. ROC = receiver operating characteristic; CT = computed tomography; HU = Hounsfield unit; MRI = magnetic resonance imaging; VBQ = vertebral bone quality.

ROC analysis showed HU outperformed VBQ in diagnostic accuracy for osteoporosis, highlighting its stronger discriminatory value (Figure 5).

## Discussion

This study evaluated the diagnostic value of CT-derived HU and MRI-based VBQ for opportunistic osteoporosis detection. Both showed independent utility, with greater potential when combined.

In our cohort, mean HU values declined across normal, osteopenia, and osteoporosis groups (158.8 ± 77.6, 117.2 ± 40.3, and 87.9 ± 49.0; p < 0.001), confirming their inverse correlation with BMD. This aligns with Schreiber et al. [10], who demonstrated a strong HU-BMD correlation, and with Pickhardt et al. [29], who proposed L1 HU <110 as a sensitive threshold, also adopted in our study and supported by Li et al. [28].

Our measured correlation coefficient between HU and DEXA T-score was r = 0.549 (p < 0.001), which, while slightly lower than Li et al. (r = 0.700 for average HU, r = 0.702 for L1 HU), still indicates a moderate-to-strong association [30]. These findings are also supported by studies such as Zou et al. [21], who demonstrated HU values were superior to DEXA T-scores in predicting pedicle screw loosening, highlighting the real-world utility of CT-based BMD assessment.

Our optimal HU threshold of ≤ 110 resulted in 87.5% sensitivity, 82.5% specificity, and an AUC of 0.90, closely matching Li et al.’s reported AUC of 0.857 for average HU and 0.850 for L1 HU. Similarly, Lee et al. [11] and Zhang et al. [20] corroborated the use of vertebral HU values as a reliable and reproducible indicator for detecting osteoporosis and assessing bone quality in spine surgery candidates.

The VBQ score, an MRI-based measurement of vertebral fat content, also increased progressively with worsening BMD in our study (3.43 ± 1.01 in normal, 3.76 ± 1.00 in osteopenia, and 4.02 ± 1.07 in osteoporosis; p = 0.047). While the correlation with T-score was weaker (r = -0.175, p = 0.065), it follows the same directional trend as observed in the study by Li et al. [30], who found r = -0.386 (average VBQ) and r = -0.413 (L1 VBQ). Our higher mean VBQ values compared to Li's (who reported 3.42 ± 0.70 for osteoporosis) may reflect technical differences in MRI scanners or variations in CSF signal reference, as noted in studies by Ehresman et al. [7,26] and Kadri et al. [27].

Our diagnostic performance metrics for VBQ (AUC = 0.75, sensitivity = 72.5%, specificity = 67.5%, threshold ≥ 3.9) exceeded those reported by Li et al. (AUC = 0.673-0.704) [29,30], and compare favorably with the findings of Ehresman et al. [31], who confirmed that VBQ independently predicts fragility fractures. Furthermore, Dieckmeyer et al. [32] and Cordes et al. [13] validated the biological relevance of bone marrow fat quantification via MRI in understanding skeletal fragility, reinforcing the mechanistic basis of VBQ scoring.

A major contribution of our study was the assessment of combination testing using HU and VBQ through series and parallel formats. In the study conducted by Li et al., combining HU and VBQ in a series format, where both thresholds had to be met, resulted in increased specificity, reaching up to 87.3%. Conversely, a parallel combination, where either threshold could be met, led to enhanced sensitivity, up to 91.5%. Our findings were consistent with this pattern. The series combination in our study, defined as HU ≤ 110 and VBQ ≥ 3.9, yielded a sensitivity of 50%, specificity of 83%, an AUC of 0.689, and a Youden index of 0.33. On the other hand, the parallel combination, defined as either HU ≤ 110 or VBQ ≥ 3.9, achieved a sensitivity of 86%, specificity of 51%, an AUC of 0.686, and a Youden index of 0.37. These results confirm the practical clinical value of using both parameters together, as HU and VBQ measure distinct aspects of bone integrity: HU reflects the mineral content of the vertebral body, while VBQ captures marrow fat composition, which is closely related to trabecular microarchitecture and bone quality, as supported by Li et al. [30], Cordes et al. [13], and Dieckmeyer et al. [32].

Our results also support the conclusions of Meredith et al. [33], who associated low HU values with postoperative fractures and adjacent segment complications, and Zou et al. [22], who found that HU predicted pedicle screw loosening more effectively than DEXA. These applications underscore the surgical relevance of HU, particularly when preoperative CT is already being acquired.

From a workflow standpoint, both HU and VBQ scores are appealing as opportunistic screening tools. Patients with lumbar degenerative disorders commonly undergo both CT and MRI as part of their routine evaluation. Incorporating HU and VBQ assessment into existing imaging protocols allows clinicians to screen for osteoporosis without additional radiation, cost, or scheduling. This is particularly useful in patients with degenerative spine disease, where DEXA can overestimate BMD due to osteophytes, disc space narrowing, or vascular calcifications [9,14].

This study’s findings are limited by its retrospective design and a relatively small sample size of 112 patients, which may affect the generalizability of the results. The study focused only on lumbar spine imaging, so it may not fully represent the overall bone health of the patient. Additionally, there was no follow-up of patients to assess long-term functional outcomes, and no clinical correlation was made with the patients’ presenting complaints. Larger prospective multicentric studies with functional correlation are required to establish the association between VBQ and DEXA values.

## Conclusions

HU values from CT and VBQ scores from MRI show promise as opportunistic tools for assessing vertebral bone quality. HU demonstrated stronger diagnostic accuracy, while VBQ offered a useful radiation-free complementary measure. These findings should be interpreted cautiously, given the retrospective design and modest sample size, and larger prospective multicenter studies are needed to validate their clinical applicability. Incorporating HU and VBQ into routine spinal imaging protocols supports earlier osteoporosis detection and better surgical decision-making, particularly in cases where DEXA is limited or unavailable.
